# Supplementary material for: Association of Maternal Smoking during Pregnancy with Neurophysiological and ADHD-Related Outcomes in School-Aged Children
Source: Int J Environ Res Public Health. 2023 Mar 7;20(6):4716. doi: 10.3390/ijerph20064716 (PMC10048892; doi:10.3390/ijerph20064716)
Supplement: Supplementary file 1 [file ijerph-20-04716-s001.zip › ijerph-2245974-supplementary.pdf]

Supplement/Additional Results

Table S1: Brain activity descriptive characteristics for EEG resting-state conditions.

		Overall N=142		Exposed n=26		Not exposed n=116		
Eyes Closed		*M*	*SD*	*M*	*SD*	*M*	*SD*	*p*
Delta	Frontal	6,52	2,23	6,47	2,12	6,53	2,27	.906
	Central	3,33	1,19	3,38	1,41	3,32	1,14	.804
	Posterior	3,27	1,31	3,45	1,54	3,23	1,26	.455
	Overall	4,47	1,41	4,53	1,55	4,46	1,39	.809
Theta	Frontal	1,53	0,72	1,36	0,74	1,57	0,71	.200
	Central	1,15	0,56	1,04	0,44	1,18	0,58	.272
	Posterior	1,31	0,68	1,27	0,72	1,32	0,67	.718
	Overall	1,35	0,61	1,24	0,62	1,37	0,60	.330
Alpha	Frontal	0,84	0,46	0,91	0,51	0,83	0,46	.434
	Central	0,73	0,41	0,84	0,46	0,71	0,40	.132
	Posterior	1,45	0,89	1,74	1,11	1,39	0,82	.066
	Overall	1,03	0,54	1,19	0,63	1,00	0,51	.153
Beta	Frontal	0,13	0,07	0,11	0,04	0,13	0,07	.014
	Central	0,08	0,04	0,07	0,03	0,09	0,05	.011
	Posterior	0,09	0,04	0,08	0,04	0,09	0,05	.399
	Overall	0,10	0,05	0,09	0,03	0,10	0,05	.024

		Overall N=142		Exposed n=26		Not exposed n=116		
Eyes open		*M*	*SD*	*M*	*SD*	*M*	*SD*	*p*
Delta	Frontal	5,46	1,82	5,23	2,00	5,51	1,78	.473
	Central	3,46	0,92	3,58	1,20	3,44	0,85	.561
	Posterior	3,45	1,18	3,55	1,56	3,43	1,08	.634
	Overall	4,19	1,15	4,17	1,46	4,19	1,07	.950
Theta	Frontal	1,09	0,43	1,10	0,65	1,09	0,37	.952
	Central	0,91	0,38	0,91	0,47	0,91	0,36	.934
	Posterior	0,88	0,38	0,87	0,44	0,89	0,37	.841
	Overall	0,97	0,36	0,96	0,49	0,97	0,33	.950
Alpha	Frontal	0,46	0,21	0,49	0,23	0,46	0,21	.520
	Central	0,45	0,25	0,53	0,29	0,43	0,24	.099
	Posterior	0,52	0,41	0,63	0,53	0,50	0,38	.162
	Overall	0,48	0,27	0,55	0,32	0,47	0,25	.165
Beta	Frontal	0,16	0,08	0,14	0,05	0,17	0,09	.068
	Central	0,10	0,05	0,09	0,03	0,10	0,05	.019
	Posterior	0,09	0,04	0,08	0,03	0,09	0,04	.272
	Overall	0,12	0,05	0,10	0,03	0,12	0,05	.069

Table S2: Association of prenatal tobacco smoking of any severity with EEG brain activity and FBB-ADHD scales among school-aged children, ***unadjusted*** for covariates.

Eyes Closed		*B*	*SE B*	*β*	R^2	*F*	*t*	*p*
Delta	Frontal	.005	.004	.110	.012	1.69	1.30	.195
	**Central**	**.004**	**.002**	**.178**	**.032**	**4.55**	**2.13**	**.035**
	**Posterior**	**.005**	**.002**	**.184**	**.034**	**4.87**	**2.21**	**.029**
	**Overall**	**.004**	**.002**	**.166**	**.028**	**3.94**	**1.98**	**.049**
Theta	**Frontal**	**.002**	**.001**	**.175**	**.031**	**4.40**	**2.10**	**.038**
	Central	.001	.001	.111	.012	1.73	1.31	.191
	**Posterior**	**.002**	**.001**	**.192**	**.037**	**5.32**	**2.31**	**.023**
	**Overall**	**.002**	**.001**	**.181**	**.033**	**4.71**	**2.17**	**.032**
Alpha	**Frontal**	**.002**	**.001**	**.223**	**.050**	**7.25**	**2.69**	**.009**
	**Central**	**.001**	**.001**	**.172**	**.030**	**4.25**	**2.06**	**.041**
	Posterior	.002	.001	.118	.014	1.97	1.40	.163
	**Overall**	**.002**	**.001**	**.178**	**.032**	**4.53**	**2.13**	**.035**
Beta	Frontal	.001	.000	.027	.001	.101	.317	.751
	Central	.001	.000	-.006	.000	.005	-.072	.942
	Posterior	.001	.000	.077	.006	.821	.906	.366
	Overall	.001	.000	.039	.002	.211	.460	.647

Eyes Open		*B*	*SE B*	*β*	R^2	*F*	*t*	*p*
Delta	Frontal	-.001	.003	-.015	.000	0.03	-0.18	.856
	Central	.002	.001	.143	.020	2.88	1.70	.092
	Posterior	.003	.002	.142	.020	2.82	1.69	.094
	Overall	.002	.002	.073	.005	0.73	0.85	.394
Theta	**Frontal**	**.001**	**.001**	**.177**	**.031**	**4.48**	**2.12**	**.036**
	**Central**	**.002**	**.001**	**.215**	**.046**	**6.70**	**2.59**	**.011**
	**Posterior**	**.002**	**.001**	**.213**	**.045**	**6.57**	**2.56**	**.011**
	**Overall**	**.001**	**.001**	**.220**	**.048**	**7.00**	**2.65**	**.009**
Alpha	Frontal	.001	.000	.153	.023	3.03	1.82	.071
	**Central**	**.001**	**.000**	**.197**	**.039**	**5.57**	**2.36**	**.020**
	Posterior	.001	.001	.102	.010	1.45	1.20	.231
	Overall	.001	.000	.152	.023	3.25	1.80	.074
Beta	Frontal	.001	.000	-.054	.003	0.40	-0.63	.529
	Central	.001	.000	.025	.001	0.09	0.30	.766
	Posterior	.001	.000	.059	.004	0.49	0.70	.485
	Overall	.001	.000	-.008	.000	0.01	-0.10	.922

FBB-ADHD	*B*	*SE B*	*β*	R^2	*F*	*t*	*p*
**Hyperactivity**	**1.26**	**.472**	**.219**	**.048**	**7.06**	**2.66**	**.009**
Attention deficit	.131	.338	.033	.001	.150	.387	.699
Impulsivity	.016	.463	.003	.000	.001	.034	.973
Total	.387	.349	.093	.009	1.23	1.11	.269

Table S3: Association of prenatal tobacco smoking of any severity with FBB-ADHD scales among school-aged children, ***adjusted*** for covariates.

FBB-ADHD	*B*	*Lower CI*	*Upper CI*	*R^2(adjusted)*	*p*	*SE*	*R^2(%)*
Hyperactivity	-.003	-.007	.001	.059	.476	.004	.600
Attention deficit	.002	-.001	.004	-.024	.599	.003	.940
Impulsivity	-.024	-.027	-.020	-.024	.896	.004	.960
Total	.000	-.004	.003	-.045	.876	.003	.950

Table S4: Significant associations between covariates included in GAMMs and outcomes.

	Brain activity	Impulsivity	Inattention	Hyperactivity	ADHD-FBB (Total)
		Higher scores = Greater problems			
Maternal measures					
↑ Age at birth	+			+	+
↑ Gestational age (week of pregnancy at birth)	+/-				
Psychopathology	+				
Smoking before pregnancy	-				
Alcohol consumption during pregnancy	-	+			+

Note. "+" = positive association, "-" = negative association, "+/-" = both positive and negative associations were observed. If there is no + or -, no significant association was observed in the GAMMs.

Table S5: Mediation Analyses* – FBB-ADHD: Hyperactivity in eyes closed resting state condition

		Smoking -> Brain activity		Brain activity-> Hyperactivity		Smoking-> Hyperactivity (direct effect)		Smoking-> Hyperactivity (indirect effect)		Total effect	
		B (95% CI)	*p*	*B* (95% CI)	*p*	*B* (95% CI)	*p*	*B* (95% CI)	*p*	*B* (95% CI)	*p*
Delta	Frontal	.006 (-.001;.013)	.092	-.012 (-.049;.026)	.545	.000 (-.001;.001)	.965	.000 (-.001;.000)	.789	-.000 (-.001;.001)	.955
	Central	**.004 (.000;.008)**	**.037**	.032 (-.034;.098)	.344	-.000 (-.002;.001)	.799	.000 (-.000;.001)	.664	-.000 (-.001;.001)	.955
	Posterior	.004 (-.001;.008)	.085	.028 (-.034;.090)	.373	-.000 (-.002;.001)	.834	.000 (-.000;.001)	.608	-.000 (-.001;.001)	.955
	Overall	**.005 (.000;.009)**	**.041**	.006 (-.053;.064)	.846	-.000 (-.001;.001)	.923	.000 (-.000;.001)	.852	-.000 (-.001;.001)	.955
Theta	Frontal	**.003 (.001;.005)**	**.009**	.010 (-.099;.120)	.852	-.000 (-.001;.001)	.915	.000 (-.000;.001)	.900	-.000 (-.001;.001)	.955
	Central	.001 (-.001;.003)	.175	.017 (-.120;.154)	.802	-.000 (-.001;.001)	.928	.000 (-.000;.000)	.860	-.000 (-.001;.001)	.955
	Posterior	.002 (-.000;.004)	.056	.032 (-.083;.146)	.586	-.000 (-.001;.001)	.873	.000 (-.000;.001)	.724	-.000 (-.001;.001)	.955
	Overall	**.002 (.000;.004)**	**.028**	.023 (-.103;.149)	.719	-.000 (.000;.004)	.892	.000 (-.000;.001)	.804	-.000 (-.001;.001)	.955
Alpha	Frontal	**.002 (.001;.004)**	**.004**	.023 (-.148;.194)	.788	.000 (-.002;.001)	.892	.000 (-.000;.001)	.848	-.000 (-.001;.001)	.955
	Central	.001 (-.000;.002)	.106	.088 (-.108;.284)	.375	-.000 (-.002;.001)	.842	.000 (-.000;.001)	.596	-.000 (-.001;.001)	.955
	Posterior	.000 (-.003;.003)	.845	.051 (-.043;.145)	.288	-.000 (-.001;.001)	.939	.000 (-.000;.000)	.952	-.000 (-.001;.001)	.955
	Overall	.001 (-.001;.003)	.171	.069 (-.083;.220)	.371	-.000 (-.001;.001)	.859	.000 (-.000;.000)	.572	-.000 (-.001;.001)	.955
Beta	Frontal	.000 (-.000;.000)	.372	-.221 (-1.391;.950)	.709	-.000 (-.001;.001)	.981	.000 (-.000;.000)	.672	-.000 (-.001;.001)	.955
	Central	.000 (-.000;.000)	.999	.198 (-1.673;2.069)	.834	-.000 (-.001;.001)	.955	.000 (-.000;.000)	.892	-.000 (-.001;.001)	.955
	Posterior	.000 (-.000;.000)	.388	.412 (-1.399;2,222)	.653	-.000 (-.001;.001)	.925	.000 (-.000;.000)	.816	-.000 (-.001;.001)	.955
	Overall	.000 (-.000;.000)	.512	-.111 (-1.796;1.574)	.896	-.000 (-.001;.001)	.962	.000 (-.000;.000)	.936	-.000 (-.001;.001)	.955

Note: *adjusted for following covariates: child's sex, child's age, maternal age at giving birth; maternal psychopathology; maternal smoking before pregnancy; maternal alcohol drinking, week of pregnancy at birth.

Table S6: Mediation Analyses* – FBB-ADHD: Hyperactivity in eyes open resting state condition

		Smoking -> Brain activity		Brain activity-> Hyperactivity		Smoking-> Hyperactivity (direct effect)		Smoking-> Hyperactivity (indirect effect)		Total effect	
		B (95% CI)	*p*	*B* (95% CI)	*p*	*B* (95% CI)	*p*	*B* (95% CI)	*p*	*B* (95% CI)	*p*
Delta	Frontal	.000 (-.006;.006)	.989	.021 (-.026;.068)	.382	-.000 (-.001;.001)	.954	.000 (-.001;.000)	.976	-.000 (-.001;.001)	.955
	Central	.003 (-.000;.006)	.084	.045 (-.042;.133)	.306	-.000 (-.002;.001)	.816	.000 (-.000;.001)	.644	-.000 (-.001;.001)	.955
	Posterior	.002 (-.002;.006)	.235	.042 (-.026;.109)	.223	-.000 (-.002;.001)	.842	.000 (-.000;.001)	.796	-.000 (-.001;.001)	.955
	Overall	.002 (-.002;.005)	371	.048 (-.028;.123)	.213	-.000 (-.001;.001)	.868	.000 (-.000;.001)	.864	-.000 (-.001;.001)	.955
Theta	Frontal	**.002 (.000;.003)**	**.011**	.040 (-.156;.236)	.689	-.000 (-.002;.001)	.874	.000 (-.000;.001)	.832	-.000 (-.001;.001)	.955
	Central	**.002 (.001;.003)**	**.004**	.020 (-.182;.223)	.842	-.000 (-.002;.001)	.908	.000 (-.000;.001)	.856	-.000 (-.001;.001)	.955
	Posterior	**.002 (.000;.003)**	**.016**	.052 (-.149;.252)	.610	-.000 (-.002;.001)	.857	.000 (-.000;.001)	.736	-.000 (-.001;.001)	.955
	Overall	**.002 (.001;.003)**	**.005**	.046 (-.172;.264)	.677	-.000 (-.002;.001)	.860	.000 (-.000;.001)	.816	-.000 (-.001;.001)	.955
Alpha	Frontal	.000 (-.000;.001)	.255	.161 (-.246;.568)	.435	-.000 (-.001;.001)	.885	.000 (-.000;.001)	.780	-.000 (-.001;.001)	.955
	Central	.001 (-.000;.001)	.221	.212 (-.111;.535)	.196	-.000 (-.002;.001)	.831	.000 (-.000;.001)	.560	-.000 (-.001;.001)	.955
	Posterior	-.000 (-.002;.001)	.493	.135 (-.065;.335)	.183	.000 (-.001;.001)	.973	-.000 (-.002;.001)	.792	-.000 (-.001;.001)	.955
	Overall	.000 (-.001;.001)	.801	.205 (-.104;.514)	.191	-.000 (-.001;.001)	.929	.000 (-.000;.000)	.852	-.000 (-.001;.001)	.955
Beta	Frontal	-.000 (-.000;.000)	.629	-.624 (-1.617;.370)	.501	-.000 (-.001;.001)	.908	.000 (-.000;.000)	.716	-.000 (-.001;.001)	.955
	Central	-.000 (-.000;.000)	.720	-.863 (-2.438;.712)	.280	-.000 (-.001;.001)	.986	-.000 (-.000;.000)	.568	-.000 (-.001;.001)	.955
	Posterior	.000 (-.000;.000)	.610	-.012 (-2.198;1.959)	.909	-.000 (-.001;.001)	.960	-.000 (-.000;.000)	.960	-.000 (-.001;.001)	.955
	Overall	-.000 (-.000;.000)	.965	-.924 (-2.530;.646)	.965	-.000 (-.001;.001)	.959	-.000 (-.000;.000)	.824	-.000 (-.001;.001)	.955

Note: *adjusted for following covariates: child's sex, child's age, maternal age at giving birth; maternal psychopathology; maternal smoking before pregnancy; maternal alcohol drinking, week of pregnancy at birth.

Table S7: Mediation Analyses* – FBB-ADHD: Inattention in eyes closed resting state condition

		Smoking -> Brain activity		Brain activity-> Inattention		Smoking-> Inattention (direct effect)		Smoking-> Inattention (indirect effect)		Total effect	
		B (95% CI)	*p*	*B* (95% CI)	*p*	*B* (95% CI)	*p*	*B* (95% CI)	*p*	*B* (95% CI)	*p*
Delta	Frontal	.006 (-.001;.013)	.092	-.024 (-.080;.033)	.405	.001 (-.001;.003)	.170	.000 (-.000;.001)	.568	.001 (-.001;.003)	.224
	Central	**.004 (.000;.008)**	**.037**	-.024 (-.080;.033)	.522	.001 (-.001;.003)	.269	.000 (-.000;.001)	.864	.001 (-.001;.003)	.224
	Posterior	.004 (-.001;.008)	.085	-.004 (-.053;.133)	.394	.001 (-.001;.003)	.278	.000 (-.000;.001)	.684	.001 (-.001;.003)	.224
	Overall	**.005 (.000;.009)**	**.041**	-.001 (-.089;.087)	.985	.001 (-.001;.003)	.215	.000 (-.000;.001)	.916	.001 (-.001;.003)	.224
Theta	Frontal	**.003 (.001;.005)**	**.009**	-.014 (-.178;.150)	.869	.001 (-.001;.003)	.196	.000 (-.000;.001)	.672	.001 (-.001;.003)	.224
	Central	.001 (-.001;.003)	.175	-.008 (-.213;.197)	.103	.001 (-.001;.003)	.218	.000 (-.000;.001)	.868	.001 (-.001;.003)	.224
	Posterior	.002 (-.000;.004)	.056	-.004 (-.053;.133)	.384	.001 (-.001;.003)	.285	.000 (-.000;.001)	.684	.001 (-.001;.003)	.224
	Overall	**.002 (.000;.004)**	**.028**	.025 (-.164;.214)	.795	.001 (-.001;.003)	.237	.000 (-.000;.001)	.980	.001 (-.001;.003)	.224
Alpha	Frontal	**.002 (.001;.004)**	**.004**	.049 (-.207;.306)	.703	.001 (-.001;.003)	.250	.000 (-.001;.001)	.960	.001 (-.001;.003)	.224
	Central	.001 (-.000;.002)	.106	.063 (-.231;.357)	.672	.001 (-.001;.003)	.247	.000 (-.000;.001)	.932	.001 (-.001;.003)	.224
	Posterior	.000 (-.003;.003)	.845	.072 (-.069;.213)	.313	.001 (-.001;.003)	.231	.000 (-.000;.001)	.980	.001 (-.001;.003)	.224
	Overall	.001 (-.001;.003)	.171	.092 (-.135;.319)	.424	.001 (-.001;.003)	.264	.000 (-.000;.001)	.756	.001 (-.001;.003)	.224
Beta	Frontal	.000 (-.000;.000)	.372	1.170 (-.568;2.909)	.185	.001 (-.001;.003)	.268	.000 (-.000;.001)	.652	.001 (-.001;.003)	.224
	Central	.000 (-.000;.000)	.999	1.530 (-1.256;4.315)	.279	.001 (-.001;.003)	.224	.000 (-.000;.000)	.948	.001 (-.001;.003)	.224
	Posterior	.000 (-.000;.000)	.388	2.445 (-.214;5.127)	.071	.001 (-.001;.003)	.281	.000 (-.000;.001)	.596	.001 (-.001;.003)	.224
	Overall	.000 (-.000;.000)	.512	2.445 (-.214;5.127)	.129	.001 (-.001;.003)	.260	.000 (-.000;.001)	.516	.001 (-.001;.003)	.224

Note: *adjusted for following covariates: child's sex, child's age, maternal age at giving birth; maternal psychopathology; maternal smoking before pregnancy; maternal alcohol drinking, week of pregnancy at birth.

Table S8: Mediation Analyses* – FBB-ADHD: Inattention in eyes open resting state condition

		Smoking -> Brain activity		Brain activity-> Inattention		Smoking-> Inattention (direct effect)		Smoking-> Inattention (indirect effect)		Total effect	
		B (95% CI)	*p*	*B* (95% CI)	*p*	*B* (95% CI)	*p*	*B* (95% CI)	*p*	*B* (95% CI)	*p*
Delta	Frontal	.000 (-.006;.006)	.989	.009 (-.062;.080)	.799	.001 (-.001;.003)	.226	.000 (-.001;.000)	.984	.001 (-.001;.003)	.224
	Central	.003 (-.000;.006)	.084	-.023 (-.155;.109)	.728	.001 (-.001;.003)	.197	-.000 (-.001;.000)	.856	.001 (-.001;.003)	.224
	Posterior	.002 (-.002;.006)	.235	.018 (-.084;.120)	.726	.001 (-.001;.003)	.239	.000 (-.000;.000)	.808	.001 (-.001;.003)	.224
	Overall	.002 (-.002;.005)	.371	.012 (-.102;.126)	.834	.001 (-.001;.003)	.231	.000 (-.001;.000)	.908	.001 (-.001;.003)	.224
Theta	Frontal	**.002 (.000;.003)**	**.011**	.166 (-.126;.458)	.261	.001 (-.001;.003)	.332	-.000 (-.000;.000)	.668	.001 (-.001;.003)	.224
	Central	**.002 (.001;.003)**	**.004**	.062 (-.241;.365)	.686	.001 (-.001;.003)	.253	-.000 (-.000;.000)	.880	.001 (-.001;.003)	.224
	Posterior	**.002 (.000;.003)**	**.016**	.101 (-.199;.400)	.507	.001 (-.001;.003)	.277	-.000 (-.000;.000)	.724	.001 (-.001;.003)	.224
	Overall	**.002 (.001;.003)**	**.005**	.137 (-.190;.463)	.408	.001 (-.001;.003)	.305	-.000 (-.000;.000)	.664	.001 (-.001;.003)	.224
Alpha	Frontal	.000 (-.000;.001)	.255	.170 (-.440;.781)	.582	.001 (-.001;.003)	.247	.000 (-.000;.001)	.996	.001 (-.001;.003)	.224
	Central	.001 (-.000;.001)	.221	.213 (-.272;.699)	.386	.001 (-.001;.003)	.264	.000 (-.000;.001)	.744	.001 (-.001;.003)	.224
	Posterior	-.000 (-.002;.001)	.493	.055 (-.246;.357)	.716	.001 (-.001;.003)	.215	.000 (-.001;.001)	.920	.001 (-.001;.003)	.224
	Overall	.000 (-.001;.001)	.801	.138 (-.328;.604)	.558	.001 (-.001;.003)	.231	.000 (-.000;.001)	.992	.001 (-.001;.003)	.224
Beta	Frontal	-.000 (-.000;.000)	.629	-.588 (-2.082;.905)	.436	.001 (-.001;.003)	.239	.000 (-.000;.001)	.900	.001 (-.001;.003)	.224
	Central	.000 (-.000;.000)	.720	-.253 (-2.624;2.118)	.833	.001 (-.001;.003)	.223	.000 (-.000;.001)	.848	.001 (-.001;.003)	.224
	Posterior	.000 (-.000;.000)	.610	.977 (-2.129;4.083)	.534	.001 (-.001;.003)	.237	.000 (-.000;.001)	.692	.001 (-.001;.003)	.224
	Overall	.000 (-.000;.000)	.965	-.425 (-2.817;1.967)	.726	.001 (-.001;.003)	.225	.000 (-.000;.001)	.952	.001 (-.001;.003)	.224

Note: *adjusted for following covariates: child's sex, child's age, maternal age at giving birth; maternal psychopathology; maternal smoking before pregnancy; maternal alcohol drinking, week of pregnancy at birth.

Table S9: Mediation Analyses* – FBB-ADHD: Impulsivity in eyes closed resting state condition

		Smoking -> Brain activity		Brain activity-> Impulsivity		Smoking-> Impulsivity (direct effect)		Smoking-> Impulsivity (indirect effect)		Total effect	
		B (95% CI)	*p*	*B* (95% CI)	*p*	*B* (95% CI)	*p*	*B* (95% CI)	*p*	*B* (95% CI)	*p*
Delta	Frontal	.006 (-.001;.013)	.092	.021 (-.034;.077)	.446	.001 (-.001;.003)	.541	.000 (-.000;.001)	.604	.001 (-.001;.003)	.465
	Central	**.004 (.000;.008)**	**.037**	**.107 (.012;.202)**	**.028**	.001 (-.001;.003)	.766	.000 (-.000;.001)	.432	.001 (-.001;.003)	.465
	Posterior	.004 (-.001;.008)	.085	**.103 (.014;.192)**	**.024**	.001 (-.001;.003)	.715	.000 (-.000;.001)	.468	.001 (-.001;.003)	.465
	Overall	**.005 (.000;.009)**	**.041**	.075 (-.010;.160)	.083	.001 (-.001;.003)	.691	.000 (-.000;.001)	.404	.001 (-.001;.003)	.465
Theta	Frontal	**.003 (.001;.005)**	**.009**	.053 (-.107;.213)	.515	.001 (-.001;.003)	.535	.000 (-.000;.001)	.596	.001 (-.001;.003)	.465
	Central	.001 (-.001;.003)	.175	.087 (-.113;.287)	.390	.001 (-.001;.003)	.535	.000 (-.000;.001)	.596	.001 (-.001;.003)	.465
	Posterior	.002 (-.000;.004)	.056	.084 (-.092;.244)	.370	.001 (-.001;.003)	.567	.000 (-.000;.001)	.596	.001 (-.001;.003)	.465
	Overall	**.002 (.000;.004)**	**.028**	.080 (-.105;.264)	.394	.001 (-.001;.003)	.576	.000 (-.000;.001)	.608	.001 (-.001;.003)	.465
Alpha	Frontal	**.002 (.001;.004)**	**.004**	.200 (-.047;.448)	.125	.001 (-.001;.003)	.760	.000 (-.000;.001)	.952	.001 (-.001;.003)	.465
	Central	.001 (-.000;.002)	.106	**.301 (.019;.583)**	**.037**	.001 (-.001;.003)	.679	.000 (-.000;.001)	.448	.001 (-.001;.003)	.465
	Posterior	.000 (-.003;.003)	.845	.080 (-.058;.217)	.225	.001 (-.001;.003)	.478	.000 (-.000;.001)	.960	.001 (-.001;.003)	.465
	Overall	.001 (-.001;.003)	.171	.182 (-.038;.402)	.104	.001 (-.001;.003)	.602	.000 (-.000;.001)	.412	.001 (-.001;.003)	.465
Beta	Frontal	.000 (-.000;.000)	.372	.641 (-1.071;2.352)	.459	.001 (-.001;.003)	.505	.000 (-.000;.001)	.540	.001 (-.001;.003)	.465
	Central	.000 (-.000;.000)	.845	.080 (-.058;.217)	.225	.001 (-.001;.003)	.478	.000 (-.000;.001)	.960	.001 (-.001;.003)	.465
	Posterior	.000 (-.000;.000)	.999	2.474 (-.225;5.173)	.072	.001 (-.001;.003)	.460	.000 (-.000;.001)	.904	.001 (-.001;.003)	.465
	Overall	.000 (-.000;.000)	.512	1.953 (-.487;4.392)	.116	.001 (-.001;.003)	.526	.000 (-.000;.001)	.404	.001 (-.001;.003)	.465

Note: *adjusted for following covariates: child's sex, child's age, maternal age at giving birth; maternal psychopathology; maternal smoking before pregnancy; maternal alcohol drinking, week of pregnancy at birth.

Table S10: Mediation Analyses* – FBB-ADHD: Impulsivity in eyes open resting state condition

		Smoking -> Brain activity		Brain activity-> Impulsivity		Smoking-> Impulsivity (direct effect)		Smoking-> Impulsivity (indirect effect)		Total effect	
		B (95% CI)	*p*	*B* (95% CI)	*p*	*B* (95% CI)	*p*	*B* (95% CI)	*p*	*B* (95% CI)	*p*
Delta	Frontal	.000 (-.006;.006)	.989	.035 (-.034;.105)	.316	.001 (-.001;.003)	.466	.000 (-.000;.001)	.920	.001 (-.001;.003)	.465
	Central	.003 (-.000;.006)	.084	**.149 (.023;.275)**	**.021**	.001 (-.001;.003)	.723	.000 (-.000;.001)	.552	.001 (-.001;.003)	.465
	Posterior	.002 (-.002;.006)	.235	.077 (-.021;.175)	.124	.001 (-.001;.003)	.576	.000 (-.001;.002)	.816	.001 (-.001;.003)	.465
	Overall	.002 (-.002;.005)	.371	.098 (-.012;.208)	.079	.001 (-.001;.003)	.559	.000 (-.000;.001)	.856	.001 (-.001;.003)	.465
Theta	Frontal	**.002 (.000;.003)**	**.011**	.241 (-.043;.524)	.095	.001 (-.001;.003)	.737	.000 (-.000;.001)	.672	.001 (-.001;.003)	.465
	Central	**.002 (.001;.003)**	**.004**	.187 (-.108;.482)	.211	.000 (-.002;.002)	.688	.000 (-.000;.002)	.544	.001 (-.001;.003)	.465
	Posterior	**.002 (.000;.003)**	**.016**	.163 (-.129;.454)	.272	.001 (-.002;.003)	.627	.000 (-.000;.001)	.604	.001 (-.001;.003)	.465
	Overall	**.002 (.001;.003)**	**.005**	.238 (-.079;.555)	.139	.001 (-.002;.002)	.732	.000 (-.000;.002)	.620	.001 (-.001;.003)	.465
Alpha	Frontal	.000 (-.000;.001)	.255	.522 (-.068;1.112)	.082	.001 (-.001;.003)	.586	.000 (-.000;.002)	.720	.001 (-.001;.003)	.465
	Central	.001 (-.000;.001)	.221	**.506 (.040;.973)**	**.034**	.001 (-.001;.003)	.625	.000 (-.000;.001)	.508	.001 (-.001;.003)	.465
	Posterior	-.000 (-.002;.001)	.493	.170 (-.124;.463)	.254	.001 (-.001;.003)	.418	.000 (-.000;.001)	.820	.001 (-.001;.003)	.465
	Overall	.000 (-.001;.001)	.801	.384 (-.067;.835)	.094	.001 (-.001;.003)	.487	.000 (-.000;.001)	.852	.001 (-.001;.003)	.465
Beta	Frontal	-.000 (-.000;.000)	.629	-.196 (-1.662;1.270)	.791	.001 (-.001;.003)	.474	.000 (-.000;.001)	.856	.001 (-.001;.003)	.465
	Central	.000 (-.000;.000)	.720	-.034 (-2.355;2.287)	.977	.001 (-.001;.003)	.466	.000 (-.000;.001)	.884	.001 (-.001;.003)	.465
	Posterior	.000 (-.000;.000)	.610	1.421 (-1.612;4.454)	.355	.001 (-.001;.003)	.494	.000 (-.000;.001)	.536	.001 (-.001;.003)	.465
	Overall	.000 (-.000;.000)	.965	.169 (-2.173;2.512)	.886	.001 (-.001;.003)	.468	.000 (-.000;.001)	.804	.001 (-.001;.003)	.465

Note: *adjusted for following covariates: child's sex, child's age, maternal age at giving birth; maternal psychopathology; maternal smoking before pregnancy; maternal alcohol drinking, week of pregnancy at birth.

Figure S1: Partial correlation matrix of EEG resting state conditions and FBB-ADHD questionnaire dimensions.

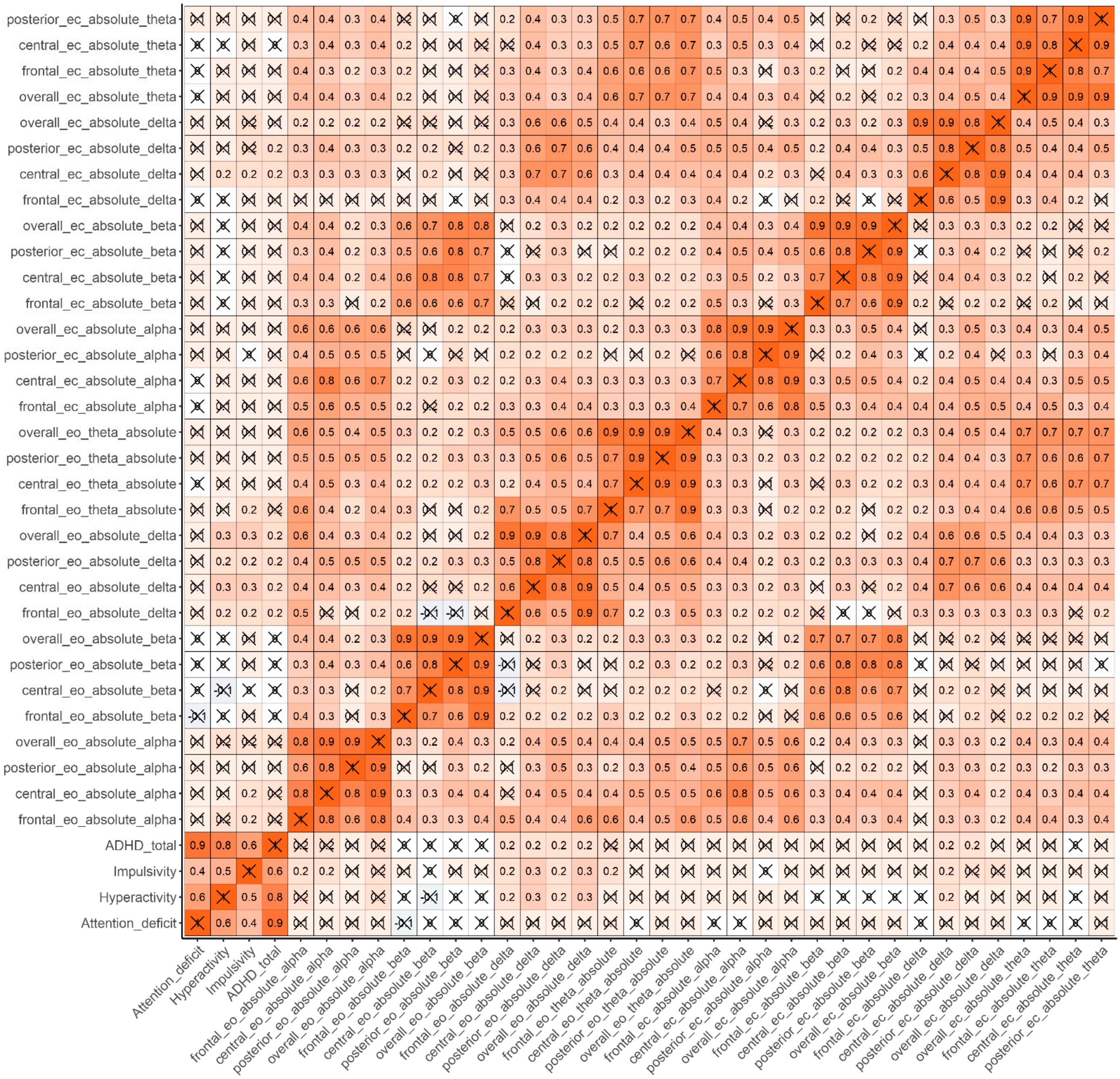

Note: X-non significant at $p < 0.05$, analysis was performed for absolute power band data (Delta 0.5-3.5 Hz, Theta 3.5-7.5 Hz, Alpha 7.5-12.5 Hz, Beta 12.5-30 Hz), included control variables: child's sex, child's age, maternal age at giving birth, week of pregnancy at birth, maternal psychopathology, maternal smoking during and before pregnancy, maternal alcohol drinking.

Figure S2: Pearson correlation matrix of Eyes open resting state condition, maternal smoking and FBB-ADHD questionnaire dimensions.

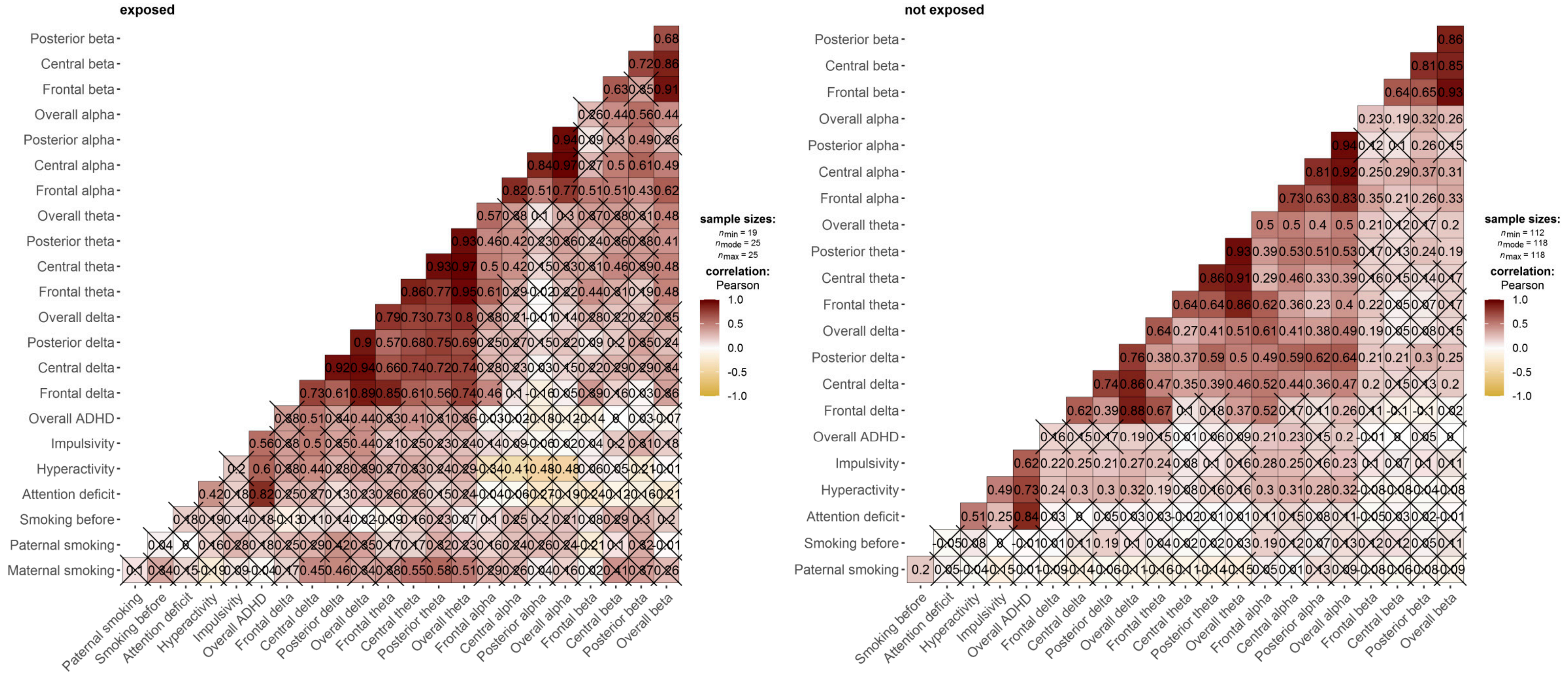

Note. X-non significant at $p < 0.05$, Parental smoking represents the averaged sum of weekly smoked cigarettes during pregnancy (Trimenon 1-3), analysis was performed for absolute power band data (Delta 0.5-3.5 Hz, Theta 3.5-7.5 Hz, Alpha 7.5-12.5 Hz, Beta 12.5-30 Hz).

Figure S3: Pearson correlation matrix of Eyes closed resting state condition, maternal smoking and FBB-ADHD questionnaire dimensions

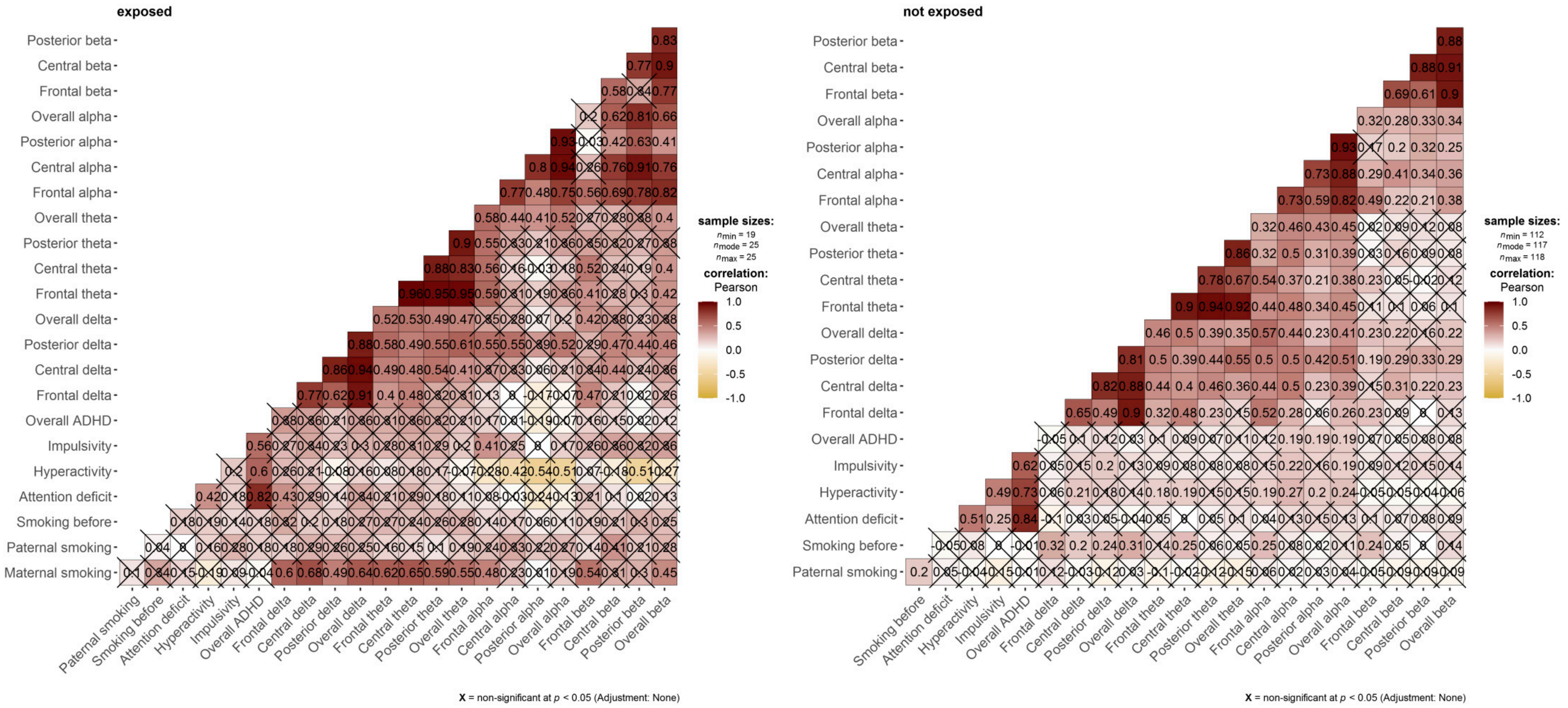

Note. X-non significant at $p < 0.05$, Parental smoking represents the averaged sum of weekly smoked cigarettes during pregnancy (Trimenon 1-3), analysis was performed for absolute power band data (Delta 0.5-3.5 Hz, Theta 3.5-7.5 Hz, Alpha 7.5-12.5 Hz, Beta 12.5-30 HZ)
